# Book Notices

**Published:** 1880-04-20

**Authors:** 


### *BOOK NOTICES.*

THE THERAPEUTICS OF GYNECOLOGY AND OBSTETRICS; Comprising the Medical, Dietetic and Hygienic Treatment of Diseases of Women, as set forth by Distinguished Contemporary Specialists. Edited by Wm. B. Atkinson, A.M., M.D., author of Hints in the Obstetric Procedure; Lecturer on Diseases of Children at the Jefferson Medical College; Physician to the Department of Obstetrics and Diseases of Women in Howard Hospital, etc., etc. Philadelphia. 1880.

This work "is the third in a series of Modern Therapeutics, originally projected by the late Dr. George H. Naphey, but which his death prevented him from completing. The work has been completed under the able supervision of Dr. W. B. Atkinson, whose wide experience in this branch of professional study is a sufficient guarantee that it has been well done."

The first two volumes of the series—*Medical Therapeutics* and *Surgical Therapeutics*—have proven so very useful and acceptable to the profession, that we doubt not the present volume will meet with prompt and favorable reception. From the examination we have made of it, we hesitate not to recommend it as a highly valuable work.

OBSERVATIONS ON AMPHORIC RESPIRATION AND AMPHORIC RESPIRATORY ECHO, by M. L. JAMES, M.D., President Richmond Academy of Medicine.

OTHER SYMPTOMS OF NERVOUS EXHAUSTION, (NEURASTHENIA), by GEORGE M. BEARD, A.M., M.D., Member of the New York Academy of Medicine, of the American Academy of Medicine, of the American Neurological Association, etc. Reprinted from the Journal of Nervous and Mental Disease, April, 1879. Chicago.

THE METRIC SYSTEM, by J. F. BALDWIN, M.D., Columbus, Professor of Anatomy, Columbus Medical College. Remarks made before the Ohio State Medical Society, June 3d, 1879, and ordered published by the Society. Columbus, O.

